# ML-driven segmentation of microvascular features during histological examination of tissue-engineered vascular grafts

**DOI:** 10.3389/fbioe.2024.1411680

**Published:** 2024-06-26

**Authors:** Viacheslav V. Danilov, Vladislav V. Laptev, Kirill Yu. Klyshnikov, Alexander D. Stepanov, Leo A. Bogdanov, Larisa V. Antonova, Evgenia O. Krivkina, Anton G. Kutikhin, Evgeny A. Ovcharenko

**Affiliations:** ^1^ Pompeu Fabra University, Barcelona, Spain; ^2^ Quantori, Cambridge, MA, United States; ^3^ Siberian State Medical University, Tomsk, Russia; ^4^ Research Institute for Complex Issues of Cardiovascular Diseases, Kemerovo, Russia

**Keywords:** histological segmentation, deep learning, vascular tissue engineering, digital pathology, tissue-engineered vascular grafts

## Abstract

**Introduction:**

The development of next-generation tissue-engineered medical devices such as tissue-engineered vascular grafts (TEVGs) is a leading trend in translational medicine. Microscopic examination is an indispensable part of animal experimentation, and histopathological analysis of regenerated tissue is crucial for assessing the outcomes of implanted medical devices. However, the objective quantification of regenerated tissues can be challenging due to their unusual and complex architecture. To address these challenges, research and development of advanced ML-driven tools for performing adequate histological analysis appears to be an extremely promising direction.

**Methods:**

We compiled a dataset of 104 representative whole slide images (WSIs) of TEVGs which were collected after a 6-month implantation into the sheep carotid artery. The histological examination aimed to analyze the patterns of vascular tissue regeneration in TEVGs *in situ*. Having performed an automated slicing of these WSIs by the Entropy Masker algorithm, we filtered and then manually annotated 1,401 patches to identify 9 histological features: arteriole lumen, arteriole media, arteriole adventitia, venule lumen, venule wall, capillary lumen, capillary wall, immune cells, and nerve trunks. To segment and quantify these features, we rigorously tuned and evaluated the performance of six deep learning models (U-Net, LinkNet, FPN, PSPNet, DeepLabV3, and MA-Net).

**Results:**

After rigorous hyperparameter optimization, all six deep learning models achieved mean Dice Similarity Coefficients (DSC) exceeding 0.823. Notably, FPN and PSPNet exhibited the fastest convergence rates. MA-Net stood out with the highest mean DSC of 0.875, demonstrating superior performance in arteriole segmentation. DeepLabV3 performed well in segmenting venous and capillary structures, while FPN exhibited proficiency in identifying immune cells and nerve trunks. An ensemble of these three models attained an average DSC of 0.889, surpassing their individual performances.

**Conclusion:**

This study showcases the potential of ML-driven segmentation in the analysis of histological images of tissue-engineered vascular grafts. Through the creation of a unique dataset and the optimization of deep neural network hyperparameters, we developed and validated an ensemble model, establishing an effective tool for detecting key histological features essential for understanding vascular tissue regeneration. These advances herald a significant improvement in ML-assisted workflows for tissue engineering research and development.

## Introduction

The development of next-generation tissue-engineered medical devices is among the leading trends in translational medicine, aimed at minimizing the invasiveness of surgical interventions and eliminating the long-term risk of device failure (McKinley et al., 2023; Subbiah, 2023). Commercial off-the-shelf tissue-engineered vascular grafts (TEVGs) and failure-protected bioprosthetic heart valves are among the upcoming game-changers in cardiovascular surgery (Fayon et al., 2021; Fioretta et al., 2021; Cho et al., 2022). The former removes the need for harvesting autologous blood vessels to construct a bypass, while the latter have an unlimited lifespan (Fayon et al., 2021; Fioretta et al., 2021; Cho et al., 2022). TEVGs have shown good implantation outcomes in sheep and have recently passed a pre-clinical trial on non-human primates, awaiting certification to commence a phase I clinical trial in Russia (Antonova et al., 2022; Antonova et al., 2023). Failure-protected bioprosthetic heart valves have demonstrated excellent resistance to enzymatic degradation and calcification *in vitro* and are also undergoing a pre-clinical trial in sheep (Kostyunin et al., 2020; 2023a; Kostyunin et al., 2023b; Kostyunin et al., 2023c; Kostyunin et al., 2023d). Although the production and distribution of these prototypes are currently limited, the high efficiency and standardized technologies of their manufacturing bode well for further development, pre-clinical testing on large animals, and eventual implementation of these innovative medical devices into routine cardiovascular surgery.

Microscopic examination is an indispensable part of animal experimentation, and hematoxylin and eosin (H&E) staining remains the most frequently used histological technique over the century (de Haan et al., 2021; Li et al., 2024). Its widespread distribution, exceptionally high clinical demand, commonly established laboratory protocols, and well-defined staining patterns have propelled the broad use of automatic slide stainers, which reduce hands-on time, and stimulated the design of machine learning (ML)-based virtual staining algorithms that curtail material expenses (de Haan et al., 2021; Li et al., 2024). Although digital pathology approaches (e.g., automated slide scanning and computer-aided analysis) are also largely integrated into the assessment of H&E-stained images (Baxi et al., 2022; Qaiser et al., 2022; Bai et al., 2023), the examination of tissue-engineered constructs is complicated by unusual and sometimes bizarre tissue architecture patterns. For instance, *de novo* formed blood vessel walls are notable for large, thin-walled capillaries resembling those assembled by endothelial cells in a 3D artificial extracellular environment, and for numerous small clusters of immune cells responsible for polymer biodegradation (Antonova et al., 2022; Antonova et al., 2023). An objective semi-quantitative analysis of regenerated microvasculature and resident polymer-digesting macrophages requires an ML-based approach to distinguish between arterioles, venules, and capillaries, to differentiate macrophage clusters from nerve trunks, and to perform adequate quantification of defined histological features.

Studies in different rat models showed that endothelial denudation achieved through balloon angioplasty triggered excessive neovascularization and promoted inflammation in the tunica adventitia and perivascular adipose tissue, also denoting an association of these processes with the development of intimal hyperplasia (Shishkova et al., 2019; Shishkova et al., 2020; Shishkova et al., 2021; Bogdanov L. et al., 2022). Intravenous administration of calciprotein particles, a trigger of endothelial dysfunction, after balloon-induced endothelial injury further exaggerated adventitial and perivascular angiogenesis, enhanced immune cell infiltration, and promoted intimal hyperplasia in rat aortas, highlighting pathophysiological links between these phenomena (Shishkova et al., 2019; Shishkova et al., 2020; Shishkova et al., 2021; Bogdanov L. et al., 2022). The saphenous vein, which has abundant *vasa vasorum* (i.e., microvessels in the tunica adventitia and adjacent adipose tissue), has been characterized by a significantly higher neointimal area in comparison with the poorly vascularized internal mammary artery (Bogdanov L. et al., 2022). Altogether, *vasa vasorum* density showed a moderate to strong correlation with leukocyte density in the tunica adventitia, and both of these parameters correlated well with the neointimal area in balloon-injured rat aortas and saphenous veins of patients undergoing coronary artery bypass graft surgery (Shishkova et al., 2019; Shishkova et al., 2020; Shishkova et al., 2021; Bogdanov L. et al., 2022).

Although the role and relative importance of neovascularization strikingly differ between pathophysiological scenarios (e.g., angiogenesis is mostly beneficial in acute ischemic conditions but is detrimental in the context of chronic inflammation), there is currently a consensus that increased amounts of *vasa vasorum* are associated with vascular inflammation and the severity of arterial stenosis in experimental models and clinical settings (Mulligan-Kehoe and Simons, 2014; Xu et al., 2015; Sedding et al., 2018; Kostyunin A. et al., 2020). Therefore, computer-assisted annotation of microvessels and immune cells in regenerated arteries may enable the discrimination of physiological and pathological patterns of vascular tissue regeneration (Weis et al., 2015; Bogdanov L. A. et al., 2022; Adamo et al., 2022; Markova et al., 2023; Timakova et al., 2023). Here, we designed a machine learning tool for the automated demarcation and quantification of blood vessels, immune cell clusters, and nerve trunks in regenerated vascular tissue that replaced biodegradable TEVGs upon their implantation into the ovine carotid artery for 6 months.

In this study, we made a significant contribution to the field of digital pathology by developing a machine learning tool capable of automated slicing of WSIs followed by delineation and quantification of blood vessels, immune cell clusters, and nerve trunks in regenerated vascular tissue. Our unique dataset, collected from a cohort of 20 sheep and comprising 104 WSIs, has been made publicly available, fostering further research. We have extensively tuned and tested various standard and state-of-the-art neural networks, proposing an ensemble approach that significantly enhances the accuracy and reliability of histological image segmentation. This work not only advances the understanding of tissue-engineered construct evaluation but also sets a new benchmark for the application of machine learning in digital pathology.

## Materials and methods

### Experimental strategy

Experimental strategy of the study is described in detail in (Antonova et al., 2022; Antonova et al., 2023). The study was conducted according to the guidelines of the Declaration of Helsinki, and was approved by the Local Ethical Committee of the Research Institute for Complex Issues of Cardiovascular Diseases (Kemerovo, Russia, protocol code 2020/06, date of approval: 19 February 2020). Animal experiments were performed in accordance with the European Convention for the Protection of Vertebrate Animals (Strasbourg, 1986) and Directive 2010/63/EU of the European Parliament on the protection of animals used for scientific purposes. For the implantation, we used female Edilbay sheep of 42–45 kg body weight which were received from the Animal Core Facility of the Research Institute for Complex Issues of Cardiovascular Diseases (Kemerovo, Russia) and selected for the surgery by Doppler ultrasonography to identify those having carotid artery diameter of 4.0 ± 0.2 mm.

Biodegradable TEVGs biofunctionalized with heparin and iloprost to prevent thrombosis (n = 20, one graft per animal) have been implanted for 6 months. Following the access to the carotid artery, we clamped it, excised a 4 cm segment, performed end-to-end implantation of a TEVG using the twisted seam (Prolene 6-0, Ethicon, Somerville, NJ, United States of America), closed the wound (Vicryl 2-0, Ethicon, Somerville, NJ, United States of America), and performed the extubation. Graft patency was assessed by Doppler ultrasonography at the baseline (immediately after the surgery), 1 day, 3 days, 1 month, 3 months, and 6 months postoperation. At the latter time point, all sheep were sacrificed. Excised TEVGs were used for the histological examination.

### Data acquisition

**FIGURE 1:**
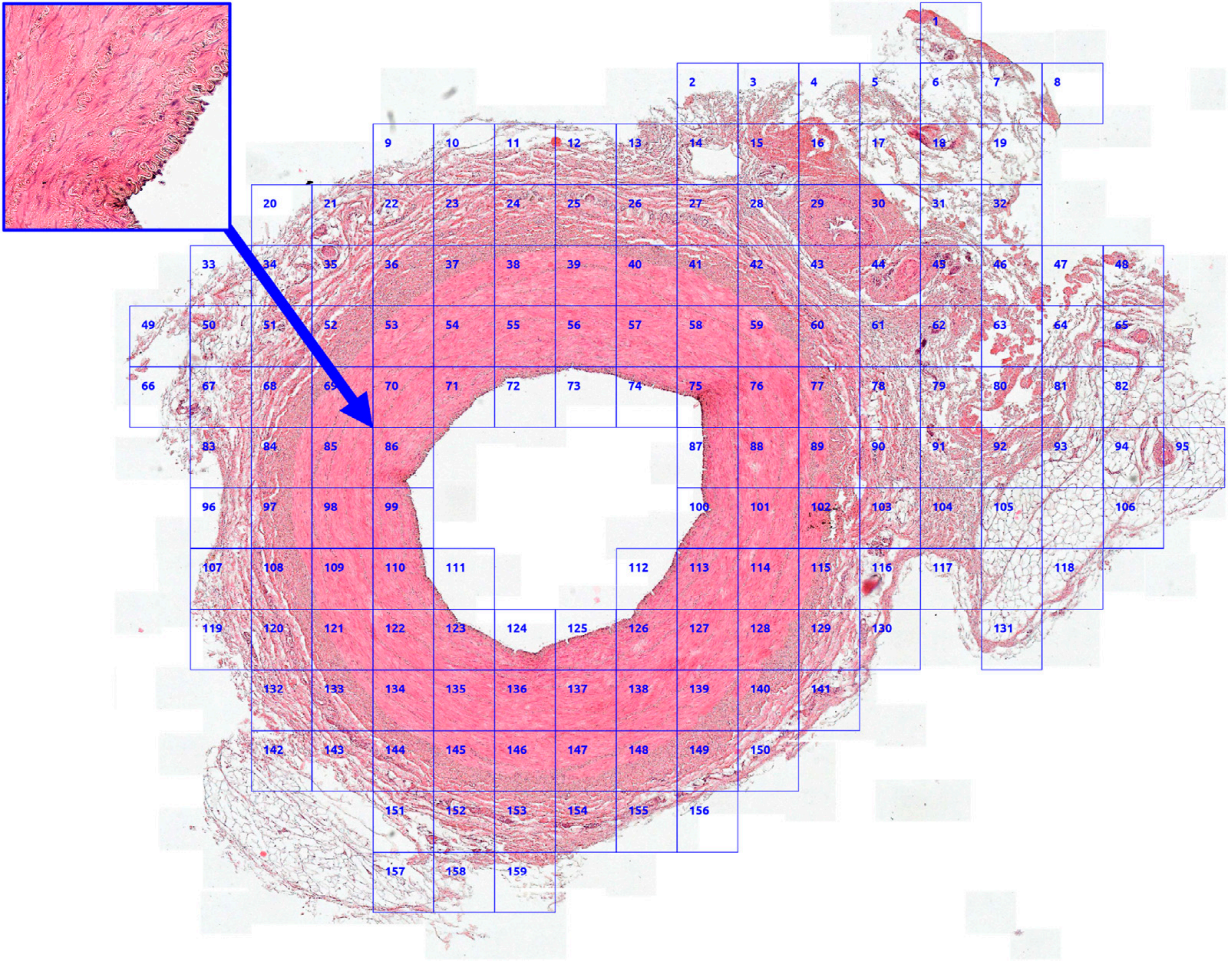
A whole slide image converted into a subset of patches obtained using Entropy Masker.

A total of 104 WSIs, each measuring an average size of 135,000 × 123,000 pixels, were obtained using an automated slide scanner (Vision Slide Assist, West Medica, Perm, Russia). The number of selected WSIs was based on achieving a balance between a sufficiently large dataset and the associated resources, thereby ensuring practical feasibility for robust model training and evaluation within the scope of our study. These WSIs were then automatically divided into 99,831 patches, each measuring 3,000×3,000 pixels. Subsequently, these patches underwent filtering using a method known as Entropy Masker, which employs specific entropy-based criteria (Song et al., 2022) to efficiently mask histology WSIs. This filtering procedure selectively retained patches containing tissue (as depicted in Figure 1), ensuring the preservation of relevant content for subsequent processing and analysis.

The Entropy Masker method addresses the task of foreground and background segmentation in WSIs, especially in scenarios where tissue structure exhibits significant porosity and heterogeneity. This approach leverages image entropy, which quantifies the randomness or complexity of pixels within a defined region or neighborhood. Initially, the method employs color hysteresis thresholding to rectify background color inconsistencies. Subsequently, the method computes the local entropy of each pixel within the image. This local entropy serves as a crucial discriminator between background noise and tissue in the WSI. By setting a threshold, the method identifies the optimal range of local entropy values to differentiate between background and tissue regions. It then assigns a Boolean value to each pixel based on a comparison of its entropy value with the threshold. These binary values are utilized to generate a tissue segmentation mask for each WSI.

**FIGURE 2:**
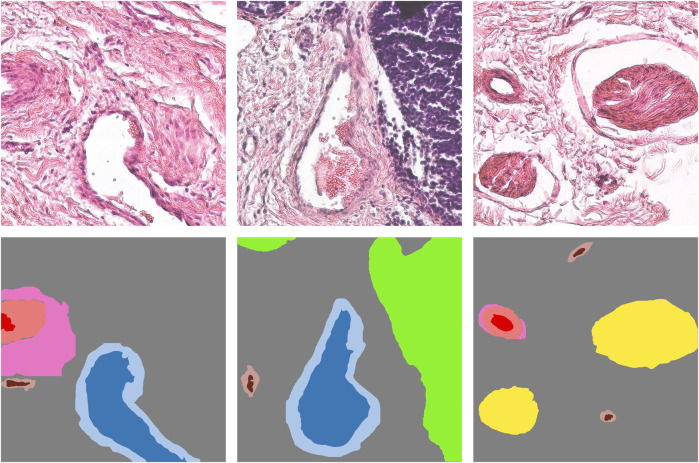
Annotation methodology for histology patches (top row) depicting features associated with a blood vessel regeneration (replacement of a biodegradable polymer by *de novo* formed vascular tissue). Histological annotations delineated with segmentation masks (bottom row) include arteriole lumen (red), arteriole media (pink), arteriole adventitia (light pink), venule lumen (blue), venule wall (light blue), capillary lumen (brown), capillary wall (tan), immune cells (lime), and nerve trunks (yellow).

After the filtering step, two pathologists performed an independent selection of 1,401 patches, aiming to balance selected histological features within the dataset, and then performed a meticulous annotation of these patches, ultimately identifying 9 histological features associated with distinct patterns of vascular tissue regeneration. These features included arteriole lumen, arteriole media, arteriole adventitia, venule lumen, venule wall, capillary lumen, capillary wall, immune cells, and nerve trunks. Each identified feature was annotated with the aid of binary masks (see Figure 2). Histology annotations were conducted using the web-based computer vision platform, Supervisely (Supervisely, 2023).

Following the initial annotation, the labeled patches were reviewed and double-verified by a senior pathologist and technical specialist responsible for dataset preparation in order to ensure the accuracy and reliability of the annotations. Verification process implied adjustments or corrections to the annotations, further enhancing their precision and consistency.

The classes described during the annotation of histological images represent different histological features of *vasa vasorum*, immune cell clusters, and nerve trunks, which are collectively responsible for the vascular tissue remodeling and adequate blood vessel response to vasoactive stimuli. A brief description of each feature is provided below:

1. Arteriole Lumen (AL): A hollow passageway through which blood flows through the arterioles, which are small vessels branching from arteries to carry oxygenated blood from the heart to the capillaries.
2. Arteriole Media (AM): A middle layer of the arterioles consisting of smooth muscle tissue and responsible for regulating blood pressure.
3. Arteriole Adventitia (AA): An outermost layer of connective tissue surrounding arterioles, providing mechanical support and protection.
4. Venule Lumen (VL): A central space within venules, which are small blood vessels that collect deoxygenated blood from the capillaries and return it to veins.
5. Venule Wall (VW): A tissue layer surrounding venules, including smooth muscle and connective tissue to regulate blood flow and maintain vessel integrity.
6. Capillary Lumen (CL): A hollow space within the capillaries, which are tiny blood vessels where oxygen and nutrients are exchanged between the blood and tissues.
7. Capillary Wall (CW): A thin layer of endothelial cells forming the capillaries and accountable for the exchange of gases, nutrients, and waste products.
8. Immune Cells (IC): Various types of cells involved in the body’s immune response, such as macrophages, lymphocytes, and neutrophils, which defend against pathogens and remove cellular debris.
9. Nerve Trunks (NT): Bundles of nerve fibers surrounded by connective tissue, responsible for transmitting nerve impulses to and from different parts of the body.

All identified features have equal importance to ensure proper regeneration. Various types of blood microvessels supply the regenerating vascular wall with oxygen and nutrients while removing metabolic waste. Nerve trunks are responsible for innervation, mediating sympathetic regulation, which adapts blood pressure through vasoconstriction and vasorelaxation. Immune cell clusters maintain local immunity and facilitate the digestion of the biodegradable polymer composing the vascular graft.

Among the microvessels, arterioles function as resistance vessels, reducing blood pressure as it flows from arteries to capillaries. Capillaries are the sites of gas and nutrient exchange, while venules collect blood from capillary beds and serve as areas for leukocyte exit from the vasculature, enabling their migration into tissues. Therefore, the term “histological feature” as used here is generally interchangeable with “histological object”. To avoid potential confusion—since the regenerating ovine carotid artery or its components might also be termed “histological objects”—we used the term “histological features” specifically for the nine defined categories. These histological feature annotations provide detailed insights into the microanatomy and cellular composition of the tissue samples, aiding in the understanding of the underlying pathological mechanisms being studied.

### Model selection

In this study, we evaluated six convolutional neural networks (CNN)—U-Net (Ronneberger et al., 2015), LinkNet (Chaurasia and Culurciello, 2017), FPN (Kirillov et al., 2017), PSPNet (Zhao et al., 2016), DeepLabV3 (Chen et al., 2017), and MA-Net (Fan et al., 2020)—for the segmentation of histological images. These models were chosen based on their established efficacy in analyzing complex biomedical images (Danilov et al., 2022).

U-Net, originally devised for medical image segmentation, enhances the basic CNN architecture by integrating a symmetric encoder-decoder structure crucial for capturing fine-grained details. Its validation in numerous studies, especially in the semantic segmentation of histological images, underscores its utility (Glänzer et al., 2023).

In addition to U-Net, we incorporated LinkNet and FPN. LinkNet is a lighter CNN that employs skip connections to reintegrate fine-grained details from the encoder to the decoder. FPN, known for its top-down architecture with lateral connections, constructs a feature pyramid. Their selection was motivated by their proven capability to delineate histologic patterns on WSIs (He et al., 2021).

PSPNet and DeepLabV3 were chosen for their adeptness in handling multi-scale feature extraction and enhancing contextual awareness. These attributes are crucial for accurately segmenting complex intravascular images, where feature sizes vary significantly and distinguishing between background and foreground can be challenging, such as in histology segmentation (Ma et al., 2018; Pan et al., 2019; Arlova et al., 2022).

Lastly, we included MA-Net, the most contemporary among the selected CNNs. By incorporating attention mechanisms into a CNN-based architecture, MA-Net directs the model’s capacity toward relevant features within an image, thereby enhancing segmentation accuracy. This model leverages the strengths of CNNs while improving feature extraction and representation.

### Hyperparameter tuning strategy

For the segmentation of the mentioned nine histological features, we meticulously selected and hypertuned six distinct segmentation networks: U-Net, LinkNet, FPN, PSPNet, DeepLabV3, and MA-Net. It is crucial to emphasize that achieving the final configurations and training settings for these networks required a rigorous process of hyperparameter tuning. Each model underwent 200 configuration trials of hyperparameter tuning to ensure optimal performance.

Our tuning process aims to maximize the segmentation score, specifically focusing on the Dice Similarity Coefficient (DSC). To achieve this goal, we utilize a DSC loss, which is calculated as follows:

$$Loss=1-\frac{2\sum \left(y_{true}\times y_{pred}\right)+\varepsilon }{\sum y_{true}+\sum y_{pred}+\varepsilon }$$ (1)

where $y_{true}$ and $y_{pred}$ represent the true and predicted label values, respectively, and ε is a small constant (set to 10^–7^ in our case) for numerical stability to avoid zero division errors.

As hyperparameter priorities vary during tuning, and certain hyperparameters have a more significant impact on network performance than others (Tobin, 2021), we focused our hyperparameter tuning efforts on specific aspects rather than trying to optimize every parameter. In particular, we did not tune hyperparameters such as batch size, nonlinearity type, optimizer options, or kernel sizes. Instead, we focused on hyperparameters that had demonstrated importance in our previous study (Danilov et al., 2022), namely, encoder architecture, input image size, optimizer selection, and learning rate. In Table 1, we provide a comprehensive summary of the hyperparameters explored during the tuning process, along with the corresponding values used.

**TABLE 1:** Hyperparameters used during the networks’ optimization.

Hyperparameter	Value	Count
Architecture	U-Net, LinkNet, FPN, PSPNet, DeepLabV3, MA-Net	6
Encoder	ResNet-18, ResNet-50, ResNet-101, MobileNet V3, EfficientNet B0, EfficientNet B3, EfficientNet B5, EfficientNet B7, RegNetX-200MF, RegNetX-6.4GF, RegNetY-12GF, SE-ResNet-50	12
Input size	512 × 512 to 896 × 896 with the step of 128 × 128 px	4
Optimizer	Adam, RAdam, SAdam, RMSprop	4
Learning rate	10^−4^, 10^−5^, 10^−6^	3

Regarding the hyperparameter search strategy, we employed Bayesian search, which, unlike Random or Grid Search, makes informed decisions. Bayesian optimization utilizes a probabilistic model to determine which values to use through an iterative process of testing values on a surrogate function before evaluating the objective function. Additionally, we utilized a specific early termination strategy, HyperBand (Li L. et al., 2016), to halt poorly performing configurations. In case of early termination, HyperBand stops the current configuration before proceeding with a new set of hyperparameter values. The combination of Bayesian optimization and HyperBand early termination forms a so-called “BOHB” (Falkner et al., 2018), an approach that offers higher computational efficiency and robustness compared to Grid Search, Random Search, or standard implementations of Bayesian optimization or HyperBand.

### Model training strategy

After conducting hyperparameter tuning and identifying optimal hyperparameters, we proceeded to train and test our models on the entire dataset we collected. To mitigate the non-uniform distribution of histological features on WSIs and address the challenge of stratified splitting, we opted for a subject-centric approach in our dataset partitioning. By adopting this approach, we aimed to enhance the reliability of our model evaluations and mitigate potential biases introduced by uneven feature distributions across WSIs.

Given the limited number of subjects studied, comprising 20 sheep, we employed a 5-fold cross-validation technique. In this approach, each fold involved 16 sheep for training and the remaining 4 for testing (Table 2; Supplementary Figure S1 of Supplementary Information). This partitioning scheme was consistently applied to maintain the integrity of subject groups within each subset. Importantly, during this process, there was no overlap between subjects in the training and testing subsets, thus preventing any form of data leakage.

**TABLE 2:** Patch and feature distributions across folds and subsets.

Fold	Subset	Patches	AL	AM	AA	VL	VW	CL	CW	IC	NT	Total
1	Train	1,168	510	512	220	675	648	770	765	409	448	4,957
	Test	233	81	84	36	186	169	178	182	91	25	1,032
2	Train	1,053	406	411	179	678	638	743	746	423	315	4,539
	Test	348	185	185	77	183	179	205	201	77	158	1,450
3	Train	1,127	507	511	222	743	702	759	760	299	423	4,926
	Test	274	84	85	34	118	115	189	187	201	50	1,063
4	Train	1,064	466	472	199	611	566	759	758	423	291	4,545
	Test	337	125	124	57	250	251	189	189	77	182	1,444
5	Train	1,192	475	478	204	737	714	761	759	446	415	4,989
	Test	209	116	118	52	124	103	187	188	54	58	1,000

During both the tuning and training steps, we employed a set of augmentation transformations using the “Albumentations” library (Buslaev et al., 2020). These augmentations not only allowed us to expand the dataset size but also served as a regularization technique, helping to mitigate overfitting during model training. The proposed augmentation workflow encompasses the following transformations:

• Horizontal flip with a probability of 50%.
• Shift, scale, and rotate with a probability of 20%: Allows for random shifts, scaling, and rotations within specified limits (shift limit = 0.0625, scale limit = 0.1, and rotate limit = 15).
• Random crop with a probability of 20%: A random-sized crop is applied with dimensions determined by a percentage of the input size, ranging from 0.8 to 0.9 times the input size.
• Conditional Padding. All images are padded to ensure a consistent size for processing.
• Gaussian noise with a probability of 20%: Adds random noise to the images with a variable intensity range, where the variance ranges from 3 to 10.
• Perspective distortion with a probability of 20%: Applies random perspective transformations to the images with a scale of 0.05–0.1.
• Random brightness and contrast adjustment with a probability of 20%: Adjusts the brightness and contrast of the images within specified limits (brightness limit = 0.2, contrast limit = 0.2).
• Hue, saturation, and value adjustment with a probability of 20%: Shifts the hue, saturation, and value of the images within specified limits (hue shift limit = 20, saturation shift limit = 30, value shift limit = 20).

In contrast to the tuning step, where a fixed batch size of 4 was utilized, the training step did not employ a fixed batch size. Since the studied models vary in complexity, they require different amounts of memory for training with a fixed batch size. Therefore, to ensure equitable training conditions, we adjusted the batch size based on the GPU memory utilization. Specifically, each model was trained with a batch size that allocated approximately 90%–100% of GPU memory.

The network training, tuning, and testing were performed on a desktop computer featuring a 16-core Intel Xeon Gold 6326 CPU @ 2.90 GHz, 128 GB of RAM, and an Nvidia A100 GPU with 40 GB of video memory. PyTorch v2.1 and Python v3.11 were utilized as the primary machine learning framework and language for network development, respectively.

## Results

### Hyperparameter tuning

Each model underwent a rigorous hyperparameter tuning process, as detailed in the Hyperparameter Tuning Strategy section, involving the examination of a total of 200 configurations. Below and in Table 3, we present the findings obtained during the tuning stage:

• Duration: The tuning time varied significantly, with FPN requiring the longest duration at 335 h and PSPNet the shortest at 187 h. The incidence of configurations crashing during the tuning process was relatively low, with MA-Net demonstrating stability by experiencing zero crashes. These crashes were typically associated with configurations demanding significant GPU memory, exceeding the available capacity.
• Encoder: The choice of encoder varied among models, with SE-ResNet-50 being the most common. However, EfficientNet B7 and RegNet variants were utilized for FPN and DeepLabV3/MA-Net, respectively.
• Input size: Input sizes were tailored to each model, ranging from 512 × 512 to 896 × 896 pixels. This variation likely reflects the trade-off between computational efficiency and the level of detail required for accurate segmentation.
• Optimizer: The optimizer of choice was predominantly RMSprop, except for U-Net, which utilized Adam, and PSPNet, which employed RAdam.
• Learning Rate: A consistent learning rate of 0.0001 was used across all models, indicating that a lower learning rate favored the convergence of these segmentation tasks.
• Accuracy: Model performance was evaluated using the DSC on a validation subset, a statistical measure of overlap between the model’s prediction and the ground truth segmentation. DSC scores ranged from 0.879 (PSPNet) to 0.906 (U-Net and FPN), indicating that U-Net and FPN achieved the highest segmentation accuracy among the tested models during the tuning stage.
• Complexity: The number of parameters and Floating-Point Operations per Second (FLOPs) offer insights into the complexity and computational demands of each model. U-Net and LinkNet were relatively similar in terms of parameters and FLOPs. However, MA-Net exhibited the highest number of parameters at 194.8 million, indicative of its complexity. DeepLabV3 recorded the highest FLOPs at 352.3 billion, suggesting it is the most computationally intensive model.

**TABLE 3:** Optimal hyperparameters for the studied networks.

Model	Encoder	Input size	Optimizer	Learning rate	Parameters, M	FLOPs, G	Configurations checked	Configurations crashed	Tuning time, h
U-Net	SE-ResNet-50	896 × 896	Adam	0.0001	35.1	128.5	190	10	231
LinkNet	SE-ResNet-50	896 × 896	RMSprop	0.0001	33.7	128.8	192	2	206
FPN	EfficientNet B7	640 × 640	RMSprop	0.0001	65.7	13.6	190	10	335
PSPNet	SE-ResNet-50	640 × 640	RAdam	0.0001	26.9	18.2	192	2	187
DeepLabV3	RegNetX-6.4 GF	768 × 768	RMSprop	0.0001	37.6	352.3	194	6	248
MA-Net	RegNetY-12GF	512 × 512	RMSprop	0.0001	194.8	119.4	200	0	248

The hyperparameter tuning stage revealed that U-Net and FPN attained the highest DSC scores, suggesting they are the most suitable models for histology segmentation on the tuning validation subset. Additionally, the stability of MA-Net with no configuration crashes and the high computational demand of DeepLabV3 are noteworthy findings.

### Model training

**FIGURE 3:**
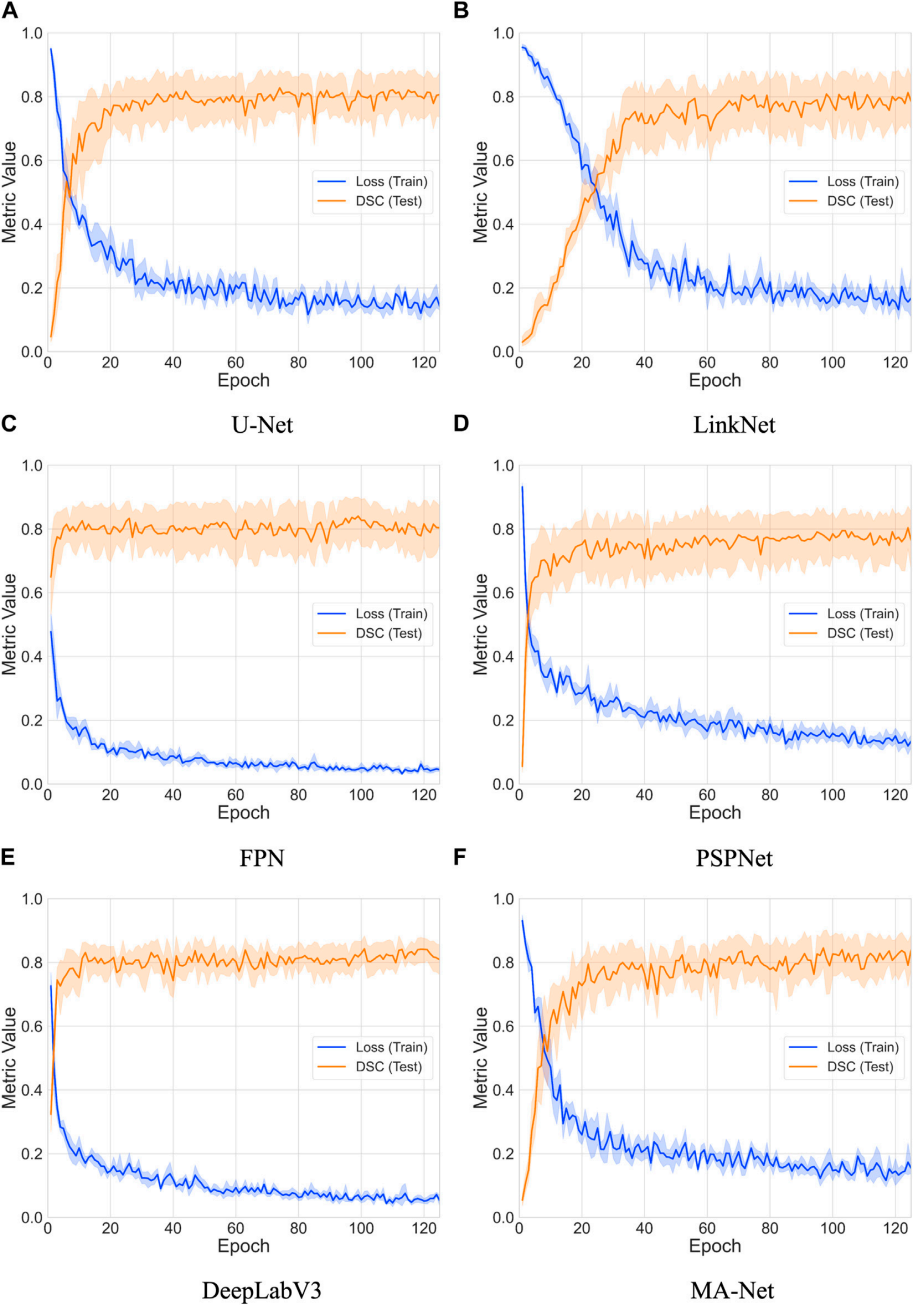
Comparative analysis of loss and DSC evolution during training and testing phases over 5-fold cross-validation with 95% confidence interval. The models compared include: **(A)** U-Net, **(B)** LinkNet, **(C)** FPN, **(D)** PSPNet, **(E)** DeepLabV3, and **(F)** MA-Net.

We conducted an extensive evaluation of the performance and convergence characteristics of six deep learning models: U-Net, LinkNet, FPN, PSPNet, DeepLabV3, and MA-Net. This comprehensive analysis spanned 125 epochs, enabling us to discern the trends in loss and Dice coefficient over time for each model (refer to Figure 3). Overall, our findings revealed a consistent pattern across all models, showcasing a gradual decrease in loss and a corresponding increase in Dice coefficient throughout the training process. These trends signify the models’ capacity to learn and enhance their segmentation capabilities as training progresses. However, it is essential to note the variations in the rate and stability of convergence observed among the different models.

To estimate convergence, we closely monitored the point at which the loss reached a stable value and when the Dice coefficient ceased to exhibit significant increases (refer to (Table 4). Convergence is typically achieved when both metrics stabilize, indicating that the model has effectively learned the segmentation task. Notably, FPN and PSPNet emerged as frontrunners in terms of convergence speed, demonstrating relatively rapid stabilization of loss and attainment of high Dice coefficients within fewer epochs. Conversely, LinkNet and MA-Net exhibited slower convergence compared to the other models, requiring a more prolonged training duration to stabilize loss and achieve comparable Dice coefficients. The observed variability in convergence speed among the models may be attributed to several factors, including architectural differences, parameter initialization techniques, and optimization algorithms. Furthermore, the utilization of diverse backbones and feature aggregation methods by the networks introduces additional complexity, potentially influencing the dynamics of convergence.

**TABLE 4:** Convergence dynamics for the models under study.

Model	Convergence, epoch	Loss convergence	DSC convergence
U-Net	110–120	0.15	0.81
LinkNet	110–120	0.15	0.80
FPN	80–90	0.04	0.82
PSPNet	90–100	0.15	0.78
DeepLabV3	90–100	0.05	0.82
MA-Net	110–120	0.15	0.82

**FIGURE 4:**
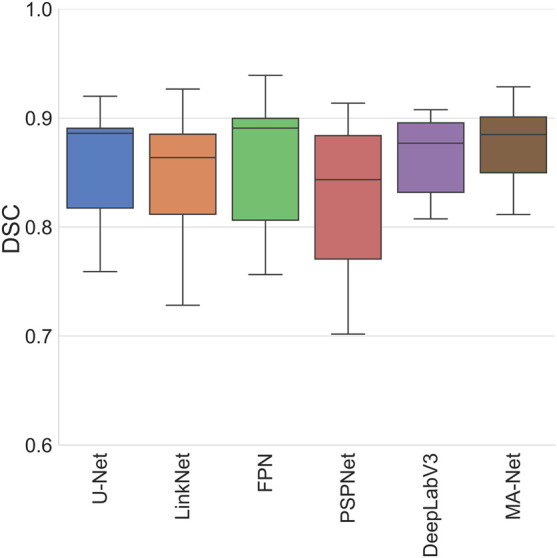
Average feature-wise performance across various models.

The outcomes of this evaluation provide valuable insights into the capabilities and limitations of the models under study (refer to Table 5 and Figure 4; Section 2 of Supplementary Information). For instance, the DeepLabV3 model exhibits robust performance across most anatomical structures, demonstrating notably high scores in venule lumen and wall as well as capillary lumen and wall segmentation. This underscores its efficacy in delineating venous and capillary structures accurately. Conversely, the FPN model showcases exceptional proficiency in segmenting immune cells and nerve trunks, indicating its aptitude for capturing intricate details within the images. On the other hand, both the LinkNet and PSPNet models exhibit variability in their performance metrics. Particularly, the PSPNet model demonstrates the lowest mean DSC of 0.823, with notably diminished scores in venule lumen, venule wall, capillary lumen, and capillary wall segmentation. These results point to inherent challenges in accurately delineating these regions. In contrast, the MA-Net architecture emerges as a frontrunner, highlighting the highest mean DSC of 0.875. It demonstrates remarkable proficiency in arteriole lumen and arteriole adventitia segmentation, suggesting its potential utility in vascular imaging applications. Lastly, the U-Net model showcases its versatility across a spectrum of anatomical structures, delivering exceptional performance in arteriole media segmentation.

**TABLE 5:** Feature-specific and mean Dice Similarity Coefficients of the studied models.

Model	AL	AM	AA	VL	VW	CL	CW	IC	NT	Mean
U-Net	0.931	**0.907**	0.820	0.797	0.766	0.801	0.783	0.920	0.966	0.855
LinkNet	0.898	0.881	0.825	0.799	0.773	0.778	0.774	0.935	0.925	0.843
FPN	0.919	0.904	0.805	0.852	0.800	0.756	0.755	**0.955**	**0.981**	0.859
PSPNet	0.872	0.838	0.830	0.784	0.734	0.728	0.722	0.937	0.959	0.823
DeepLabV3	0.872	0.861	0.803	**0.900**	**0.861**	**0.815**	**0.793**	0.895	0.975	0.864
MA-Net	**0.939**	0.893	**0.860**	0.848	0.830	0.806	0.787	0.937	0.978	**0.875**

Bold values highlight the model that performed best for each specific feature.

**FIGURE 5:**
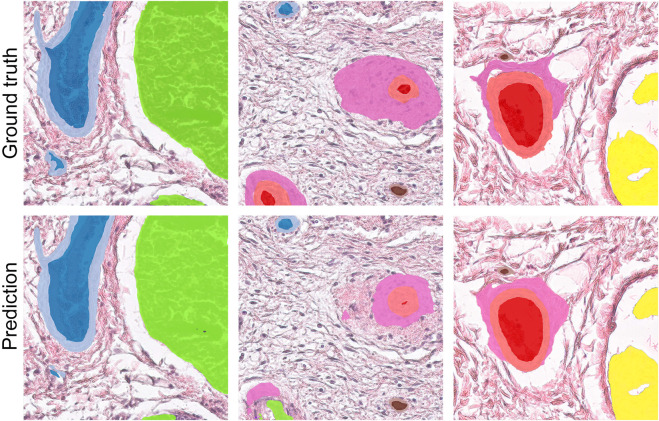
Comparison between ground truth segmentation and ensemble predictions.

To illustrate the network predictions, we provide three patches showcasing the segmentation of the studied histologic features in Figure 5. This figure presents predictions derived from an optimal solution: an ensemble of three models (MA-Net, DeepLabV3, and FPN). Further discussion on this ensemble is provided in the subsequent Discussion section. In addition, a comprehensive visualization of all models used in our study can be found in Supplementary Figures S3, S4 of Supplementary Information.

## Discussion

### Optimizing model selection for segmentation

**FIGURE 6:**
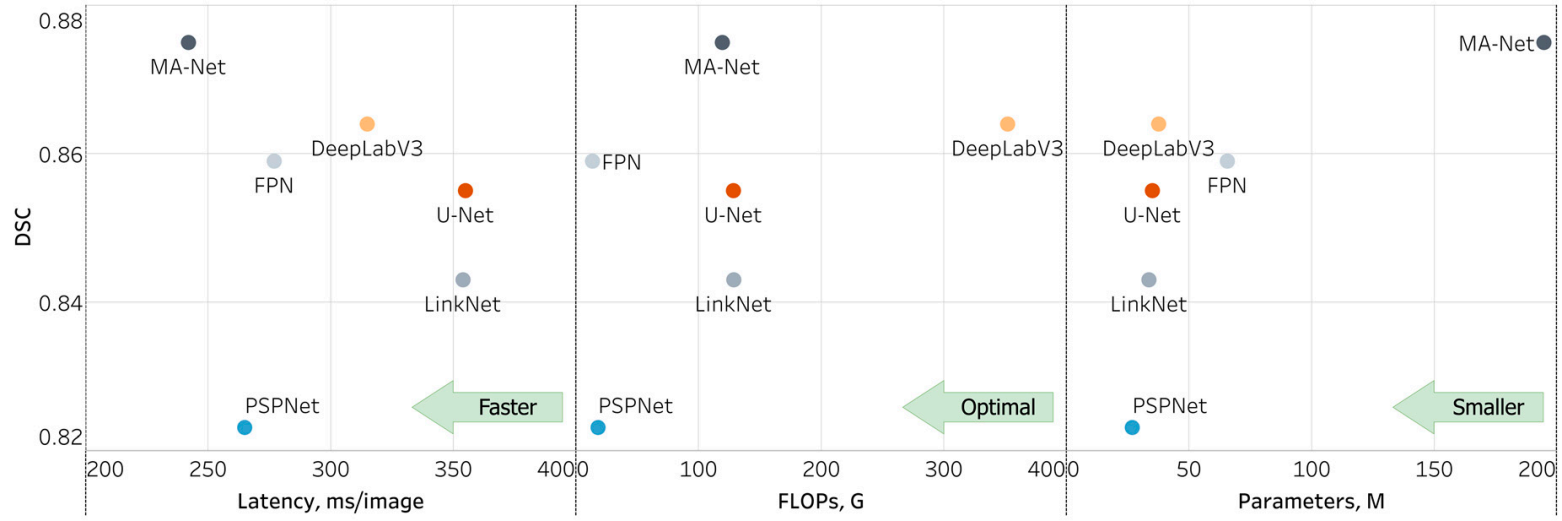
Comparison of models for microvascular segmentation in tissue-engineered vascular grafts.

The variability in model performance (refer to Figure 6) across different biological structures highlights the critical need for tailored model selection, based on the specific requirements of the segmentation task at hand. This is particularly pertinent in the field of medical imaging, where precise segmentation of various anatomical structures is essential for accurate diagnosis and treatment planning. If processing time is not a critical factor for a given task, we recommend employing an ensemble of three models: MA-Net for the segmentation of arteriole structures, DeepLabV3 for venous and capillary structures, and FPN for the segmentation of immune cells and nerve trunks. This ensemble achieves an average DSC of 0.889, surpassing the DSCs of MA-Net (0.875), DeepLabV3 (0.864), and FPN (0.859) when used individually. While ensembling leads to improved segmentation performance, it also results in a threefold increase in processing time—from an average model processing speed of 278 ms/image to 911 ms/image with the ensemble. It is important to note that this time pertains to the segmentation of a patch rather than an entire WSI. Consequently, the overall processing time for a WSI increases significantly. For example, processing a WSI comprising 159 patches, as shown in Figure 1, with this ensemble could be a time-consuming operation, taking approximately 145 s, compared to roughly 38 s when only MA-Net is utilized.

### Recommendations for performance enhancement

While each model has its strengths and weaknesses, focusing on computational efficiency, parameter optimization, and the balance between latency and accuracy can significantly enhance performance. Tailoring these strategies to the specific requirements and constraints of the medical imaging task will be crucial in optimizing the model’s efficacy. Below we outline several methodological steps that should help improve performance:

• Computational Efficiency: For models like DeepLabV3 with high FLOPs, adopting techniques such as pruning, quantization, or knowledge distillation could reduce the computational load without significantly compromising accuracy (Li H. et al., 2016; Krishnamoorthi, 2018; Gou et al., 2021; Kim et al., 2021).
• Parameter Optimization: Models with a high number of parameters, such as MA-Net, could benefit from regularization techniques or architecture adjustments to reduce the risk of overfitting and improve generalization.
• Balancing Latency and Accuracy: Implementing multi-scale architectures or attention mechanisms could help models like PSPNet improve accuracy without a substantial increase in latency or computational cost (Huang et al., 2019).
• Model Simplification: For applications where real-time performance is critical, simplifying the model architecture or employing lightweight versions of existing models (e.g., MobileNetV3 as a backbone) could offer a good trade-off between speed and accuracy (Howard et al., 2019).
• Advanced Training Techniques: Utilizing more sophisticated training strategies like learning augmentation policies (refer to AutoAugment), self-supervised or semi-supervised learning could enhance the model’s ability to generalize and improve its segmentation accuracy (Kingma et al., 2014; Cubuk et al., 2018; Zhou et al., 2018; Krishnan et al., 2022; Rani et al., 2023).

### Limitations and perspectives

The comparative study of deep learning models for histological image segmentation reveals varying degrees of effectiveness across different architectures. Models like MA-Net, DeepLabV3, and FPN have demonstrated superior performance in segmenting intricate details within histological images, underscoring the potential of deep learning in refining diagnostic processes and treatment outcomes. These findings suggest a promising future for the integration of deep learning models into various pre-clinical and clinical settings, potentially accelerating histological analysis of digital images, improving the precision of digital pathology diagnostics and enabling tailored treatment strategies. However, challenges such as the need for extensive labeled datasets and significant computational resources remain. Besides that, the scope can be broadened to include additional histological features characterizing regeneration of vascular tissue or other tissues and organs, to extend the list of tissue-engineered constructs which are currently under development, and to analyze other pre-clinical animal models such as swine or non-human primates. Furthermore, our networks were trained to discriminate between distinct layers of the vascular wall, e.g., tunica media and tunica adventitia. This suggests their potential applicability for the analysis of pathologies characterized by microvasculature remodeling such as pulmonary arterial hypertension, where lung arterioles suffer from intimal, medial, and adventitial thickening (Tuder, 2017; Tobal et al., 2021; Jia et al., 2023). Therefore, the results of our study are not limited to the fields of tissue engineering and regenerative medicine, and can be used in pathophysiology to analyze the results of disease modeling, as well as in medical pathology to improve diagnosis after clinical validation.

### Compatibility with 3D imaging modalities

The development, testing, and validation of three-dimensional imaging modalities, which may include optical clearing of entire tissue segments, are among the current trends in modern histology. For instance, the light-sheet imaging technique, which employs a non-destructive and sectioning-free approach, enables both planar and volumetric microscopy. This technique is a promising diagnostic tool that is also compatible with AI-assisted blood vessel annotation (Glaser et al., 2017; Glaser et al., 2019; Glaser et al., 2022). An important advantage of open-top light-sheet microscopy is its ability to conduct wide-area microscopy on uneven surgical specimens, perform volumetric dual-channel imaging of core-needle biopsies, and facilitate rapid intra-operative microscopy of the interface between the tumor and intact tissue.

In terms of machine learning applications, light-sheet microscopy has been coupled with algorithms for intensity leveling and digital staining, also known as virtual staining or false-coloring (Serafin et al., 2020). Additionally, it has been integrated with prognostication software based on weakly supervised learning, capable of predicting clinical outcomes in patients with prostate cancer (Song et al., 2024). Recently, light-sheet imaging has been combined with an algorithm for automated vasculature analysis, enabling the quantification of blood vessel trees in various organs after staining with fluorescein isothiocyanate-labeled albumin and optical clearing (Spangenberg et al., 2023).

Several other studies have utilized ML for the automated quantification of blood vessel trees in intact tissue segments following optical clearing (Kostrikov et al., 2021; Takahashi et al., 2022; Yang et al., 2024), as summarized in a critical review (Zhu et al., 2021). However, these studies focused on the automated recognition of blood vessel trees rather than individual blood vessels, which contrasts with the approach used in our study. While the delineation of blood vessel trees in 3D imaging after optical clearing offers more accurate vasculature quantification due to its volumetric capabilities, it does not distinguish between different types of microvessels (i.e., arterioles, venules, and capillaries), discern vessel lumen from the vessel wall, or differentiate between the layers of the vessel wall. This limitation significantly restricts its application in histology and vascular pathophysiology. Ideally, both approaches should be used in conjunction for a comprehensive analysis of vascularization.

### Integration with virtual staining and stain transformation techniques

A promising trend in contemporary histology is the development and implementation of virtual staining technology, which involves digitally staining label-free histological images or transforming images between various histochemical stains (de Haan et al., 2021; Zhang et al., 2022; Latonen et al., 2024). An ideal histology workflow would include virtual staining of deparaffinized or frozen sections, with the ability to switch between common stains such as H&E, Masson’s trichrome (including modification with Verhoeff’s stain), van Gieson’s (including modification with Weigert’s elastic stain), periodic acid-Schiff, Picrosirius Red, and Movat’s pentachrome. This would be followed by ML-driven annotation of histological features of interest, such as blood vessels.

Training these neural networks should involve the same tissue section after staining, adjacent sections at various distances (e.g., 50, 100, 200, 500, and 1,000 μm), and sections from different tissues to perform multi-step cross-validation of the ML tools. Both supervised and unsupervised training strategies can be employed for this task (Abraham and Levenson, 2024; Alajaji et al., 2024; Pillar et al., 2024).

Current generative staining models, especially CycleGAN, have shown results nearly equivalent to actual staining, though supervised learning models (such as pix2pix or conditional GAN) generally demonstrate better metric values than unsupervised approaches (Bai et al., 2023; Salido et al., 2023; Latonen et al., 2024). However, due to the wide range of organisms, tissues, and staining varieties, ML strategies show varying and sometimes contradictory results when applied to virtual staining or stain-to-stain transformation tasks. Nevertheless, the definition of blood vessels might be more robust due to their common geometrical patterns and similar histological structures (e.g., an endothelial cell layer) across species.

Transforming H&E images into specific vascular stains (e.g., a combination of Masson’s trichrome with Verhoeff’s, van Gieson’s with Weigert’s, or Movat’s pentachrome) could improve the accuracy of ML-mediated blood vessel annotation and increase the Dice similarity coefficient, a key metric for this task. Additionally, virtually transforming H&E or vascular-specific stains into immunohistochemical stains in initially unstained images holds promise, particularly for recapitulating canonical protein markers (e.g., CD31 for endothelial cells, type IV collagen for basement membrane, SM-MHC or α-SMA for vascular smooth muscle cells, and CD45 for leukocytes).

As blood vessels have well-defined markers (CD31, SM-MHC, α-SMA) and intravascular structures (type IV collagen and elastin), virtual immunohistochemistry could be especially useful in vascular research. This is supported by a superior Dice similarity coefficient documented in arterioles, which are discernible from venules and capillaries even with H&E staining because of dense internal elastic lamina, as demonstrated in our study.

In conclusion, there is a consensus that optimizing neural network architecture is an effective strategy for improving the accuracy of virtual staining. This will enhance the credibility of this approach and maximize its potential for implementation in tissue engineering, pre-clinical trials, and clinical practice (Bera et al., 2019; Khan et al., 2023).

### Cross-species versatility

The applicability of ML-based models to a wide range of animal species is fundamental to their integration into pathophysiological studies and the development of tissue-engineering medical devices. Previously, ML algorithms have demonstrated their efficiency in high-throughput measurements of vascular density in phase contrast and fluorescent images of 3D tissue-engineered constructs derived from human and rat adipose tissue (Strobel et al., 2021). In this study, ML-based software was able to annotate blood vessels in a 3D scaffold, further calculating blood vessel length and density within a given area to evaluate angiogenesis. The authors highlighted that the rapidity and accuracy of such vascular growth monitoring software are key advantages for its integration into the manufacturing of 3D tissue-engineered constructs. Furthermore, it has been suggested that this software can be improved to identify other histological parameters, such as cellular density.

In another study, an ML-based algorithm was employed to analyze three immunofluorescence image datasets: rat abdominal wall (artificial defect closed with a VEGF-loaded elastomeric patch), rat infarcted heart treated with an electrospun extracellular matrix-enriched polymer patch, and mouse metanephric kidneys transplanted into the omentum of recipient mice (Adamo et al., 2022). The algorithm was capable of quantifying the number and size of blood vessels with comparable performance across all datasets. Although these results demonstrated the versatility of the blood vessel-annotating algorithm for different tissue sources and tissue-engineered constructs, the authors noted its incompatibility with H&E or Masson’s trichrome staining images.

Recently, an ML-based tool was developed to quantify vascularization in thick sections of mouse hearts using a 3D histology approach (Lapierre-Landry et al., 2023). This tool annotated blood vessels through 4′,6-diamidino-2-phenylindole nuclei staining and autofluorescence patterns. In addition to vessel length density, the tool also calculated the median distance from the nearest vessel, which is informative for studying hypoxia in cancer tissues. The authors suggested that their algorithm could be adapted to segment any structure in any organ of interest using the 3D histology approach.

ML-driven algorithms for the segmentation of hematoxylin- and chromogen-stained blood vessels have also been employed in the pathophysiological modeling of Alzheimer’s disease (Bukenya et al., 2020) and pre-clinical trials, as presented in our study.

Thus, there is a consensus that ML approaches are versatile and can be applied to different species and organs (Komura and Ishikawa, 2018; Timakova et al., 2023). In clinical practice, deep learning models for blood vessel segmentation have been utilized to define vascularization through specific protein markers (CD31, CD34, and type IV collagen), delineating vessel geometry (Kather et al., 2015; Karageorgos et al., 2024). However, the latter study exploited a relatively expensive, time-consuming, and technically challenging immunofluorescence staining technique, which is rarely used compared to H&E staining, and the Dice similarity coefficient did not exceed 0.71 (Karageorgos et al., 2024). Automated ML-assisted annotation of H&E-stained images has been successfully implemented for the detection of microvessels in patients with lung cancer (Yi et al., 2018), breast cancer (Chen et al., 2023), and glioma (Li et al., 2019), with ML-defined microvessel density being associated with patient survival rates (Yi et al., 2018; Li et al., 2019).

## Conclusion

The study demonstrates the efficacy of machine learning in the segmentation of histological images as well as in the annotation of distinct types of microvessels, immune cell clusters, and nerve trunks, thus providing a ready-to-use solution to evaluate tissue-engineered medical devices for cardiovascular surgery. It is crucial to highlight the importance of selecting and tuning machine learning models for specific segmentation tasks, as this can significantly enhance the accuracy of histological analysis. In addition, the ensemble model combining MA-Net, DeepLabV3, and FPN was shown to outperform individual segmentation networks, resulting in a mean DSC of 0.889. This provides a robust tool for precise analysis of the abovementioned histological features which define the development of physiological and pathophysiological scenarios during vascular tissue regeneration. This advancement holds significant implications for translational medicine, offering a pathway to rapid improvement and implementation of next-generation tissue-engineered constructs into regenerative medicine. It is worth noting that, the public release of our dataset further contributes to the field, enabling ongoing research and development. Future efforts will focus on enhancing computational efficiency and exploring the clinical applicability of our findings, with the ultimate goal of integrating these technologies into routine practice in digital pathology and translational medicine.

## Data Availability

The data supporting the key findings of this study are presented within the article/Supplementary material. All essential components of the study, including curated source code, data, and trained models, have been made publicly available: Source code: https://github.com/ViacheslavDanilov/histology_segmentation. Dataset: https://doi.org/10.5281/zenodo.10838384. Models: https://doi.org/10.5281/zenodo.10838431.
