# Supplementary material for: ML-driven segmentation of microvascular features during histological examination of tissue-engineered vascular grafts
Source: Front Bioeng Biotechnol. 2024 Jun 26;12:1411680. doi: 10.3389/fbioe.2024.1411680 (PMC11233802; doi:10.3389/fbioe.2024.1411680)
Supplement: Supplementary file 1 [file Presentation1.pdf]

SUPPLEMENTARY INFORMATION

ML-Driven Segmentation of Histological Features During Microscopic Examination of Tissue-Engineered Vascular Grafts

Viacheslav V. Danilov, *PhD* [1,2,*], Vladislav V. Laptev, *MSc* [3,4], Kirill Yu. Klyshnikov, *MD, PhD* [4], Alexander D. Stepanov, *BSc* [4], Leo A. Bogdanov, *PhD* [4], Larisa V. Antonova, *MD, DSc* [4], Evgenia O. Krivkina, *MSc* [4], Anton G. Kutikhin, *MD, DSc* [4], and Evgeny A. Ovcharenko, *PhD* [4]

[1] *Pompeu Fabra University, Barcelona, Spain*

[2] *Quantori, Cambridge, Massachusetts, United States*

[3] *Siberian State Medical University, Tomsk, Russia*

[4] *Research Institute for Complex Issues of Cardiovascular Diseases, Kemerovo, Russia*

E-mail: viacheslav.v.danilov@gmail.com (Viacheslav V. Danilov)
* Corresponding author

CONTENTS:

1. Data distribution for model training
2. Model performance on the test subset
3. Comparison of model predictions with ground truth annotation

1. Data distribution for model training

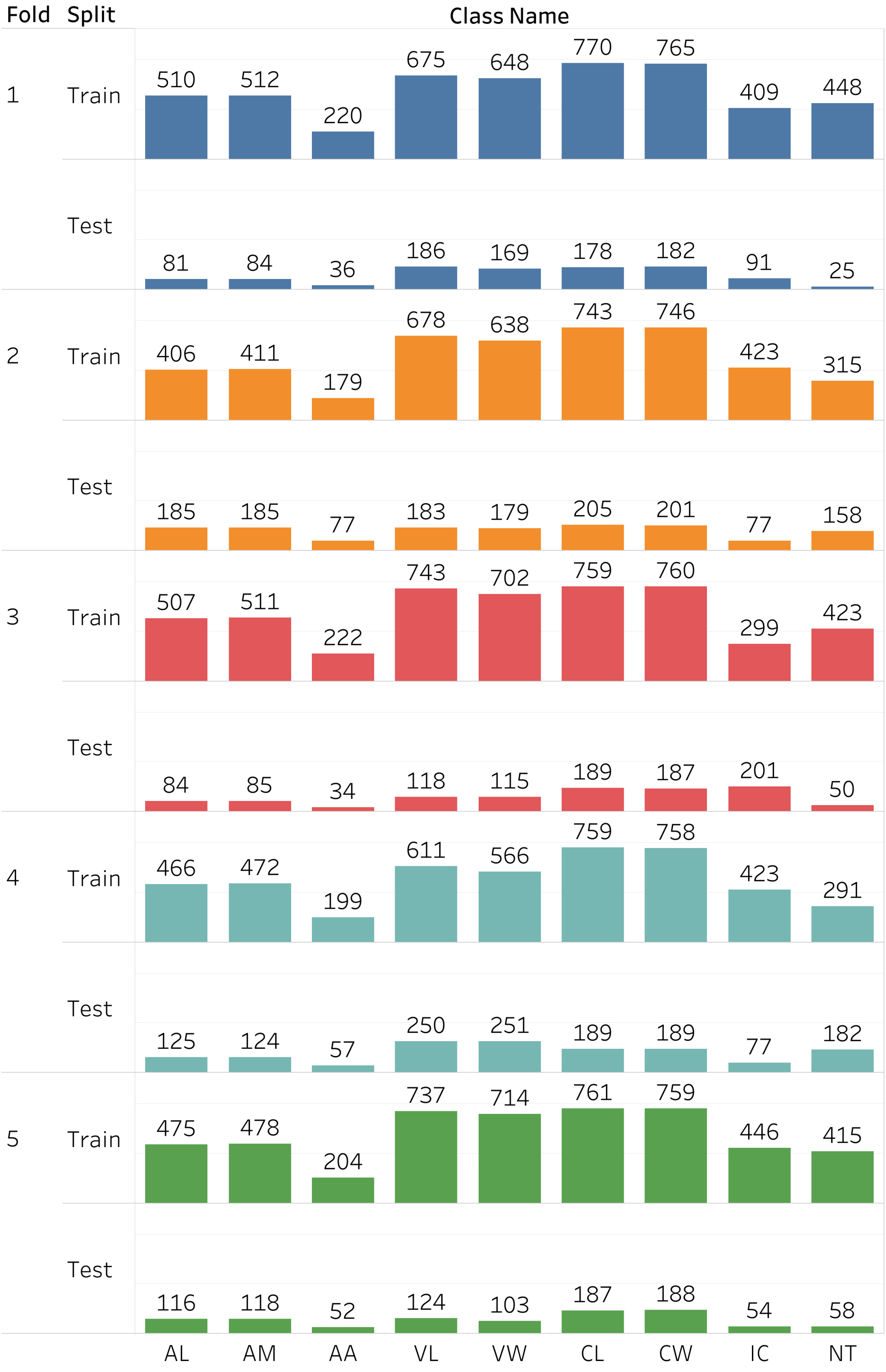

Figure S1. *Feature-wise distribution of histological features across folds and subsets for model training and testing.*

2. Model performance on the test subset

Table S1. *DSC values on the test subset across different folds at optimal epoch.*

Model	Fold	Epoch	AL	AM	AA	VL	VW	CL	CW	IC	NT	Mean
U-Net	1	46	0.945	0.913	0.909	0.835	0.772	0.871	0.866	0.957	0.949	0.891
	2	38	0.858	0.860	0.690	0.837	0.831	0.775	0.717	0.844	0.946	0.817
	3	88	0.940	0.868	0.765	0.607	0.585	0.596	0.565	0.909	0.997	0.759
	4	68	0.972	0.961	0.852	0.776	0.755	0.882	0.886	0.912	0.979	0.886
	5	108	0.942	0.935	0.884	0.928	0.888	0.882	0.881	0.979	0.961	0.920
LinkNet	1	95	0.913	0.880	0.901	0.866	0.764	0.856	0.853	0.954	0.981	0.885
	2	72	0.738	0.754	0.845	0.879	0.867	0.671	0.628	0.954	0.970	0.812
	3	114	0.932	0.880	0.688	0.582	0.651	0.590	0.640	0.884	0.708	0.728
	4	82	0.965	0.950	0.816	0.734	0.692	0.867	0.858	0.911	0.980	0.864
	5	104	0.942	0.942	0.875	0.936	0.891	0.904	0.891	0.974	0.986	0.927
FPN	1	98	0.894	0.870	0.676	0.899	0.863	0.930	0.920	0.971	0.995	0.891
	2	63	0.898	0.890	0.715	0.788	0.775	0.636	0.652	0.978	0.925	0.806
	3	25	0.919	0.888	0.771	0.764	0.722	0.422	0.419	0.904	0.998	0.756
	4	113	0.949	0.939	0.902	0.849	0.723	0.902	0.899	0.943	0.992	0.900
	5	80	0.937	0.934	0.961	0.959	0.916	0.889	0.886	0.978	0.993	0.939
PSPNet	1	60	0.969	0.925	0.943	0.816	0.708	0.848	0.837	0.961	0.949	0.884
	2	88	0.671	0.645	0.781	0.836	0.799	0.663	0.611	0.967	0.963	0.771
	3	124	0.870	0.846	0.652	0.675	0.584	0.415	0.441	0.878	0.955	0.702
	4	100	0.919	0.861	0.865	0.660	0.698	0.841	0.849	0.913	0.986	0.844
	5	111	0.929	0.916	0.909	0.930	0.883	0.874	0.873	0.968	0.941	0.914
DeepLabV3	1	112	0.930	0.921	0.964	0.841	0.769	0.857	0.856	0.955	0.969	0.896
	2	50	0.698	0.695	0.939	0.882	0.858	0.653	0.622	0.928	0.993	0.807
	3	50	0.926	0.896	0.802	0.907	0.898	0.886	0.884	0.704	0.988	0.877
	4	85	0.919	0.888	0.424	0.920	0.882	0.811	0.747	0.959	0.937	0.832
	5	109	0.887	0.903	0.887	0.951	0.900	0.868	0.854	0.931	0.989	0.908
MA-Net	1	98	0.976	0.940	0.933	0.799	0.795	0.869	0.863	0.971	0.964	0.901
	2	32	0.896	0.878	0.876	0.852	0.856	0.740	0.660	0.931	0.961	0.850
	3	118	0.920	0.822	0.758	0.851	0.838	0.628	0.606	0.882	0.998	0.812
	4	93	0.946	0.893	0.863	0.809	0.754	0.895	0.904	0.925	0.974	0.885
	5	102	0.956	0.931	0.870	0.928	0.905	0.899	0.905	0.976	0.990	0.929

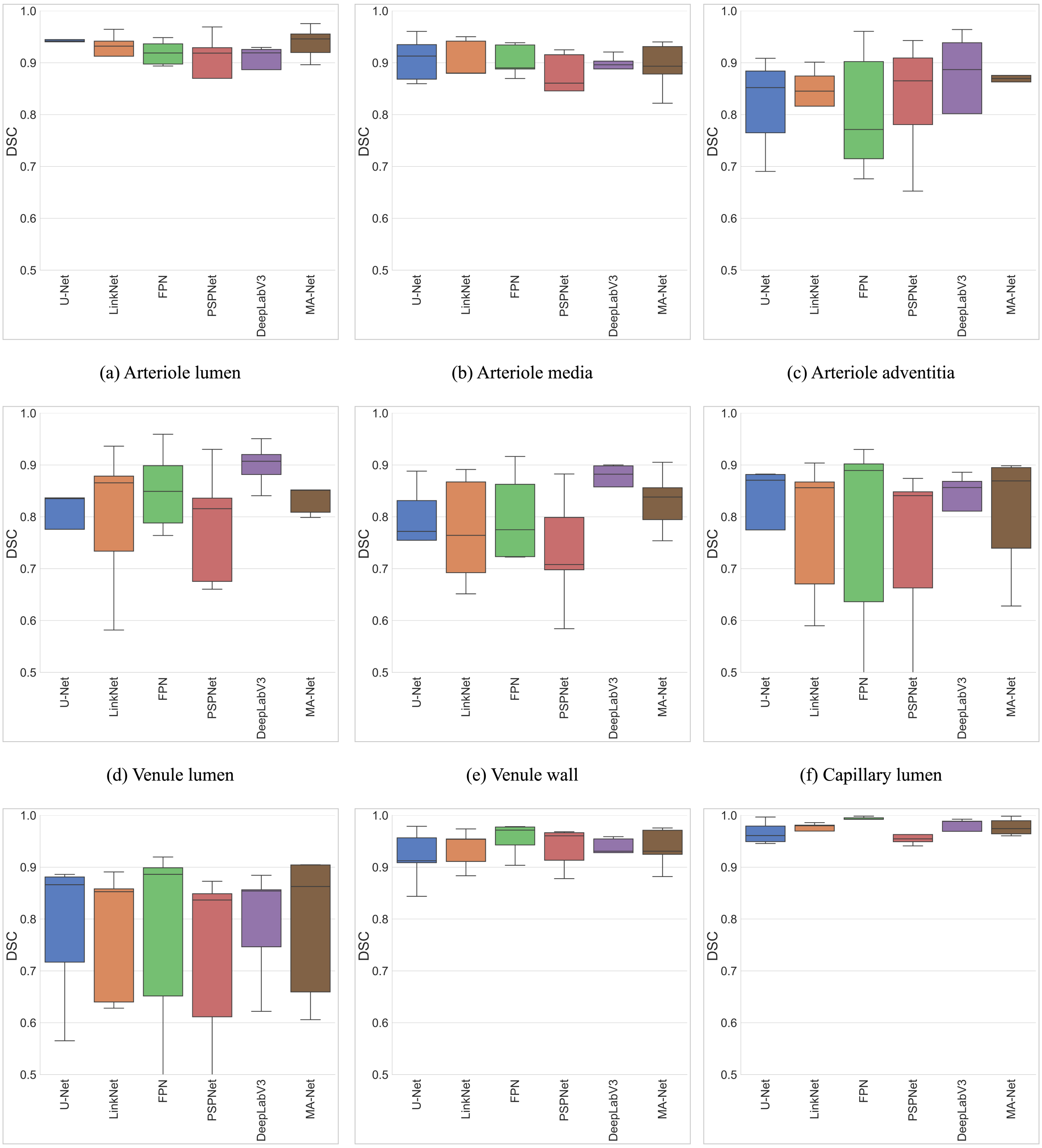

(a) Arteriole lumen (b) Arteriole media (c) Arteriole adventitia

(d) Venule lumen (e) Venule wall (f) Capillary lumen

(g) Capillary wall (h) Immune cells (i) Nerve trunks

Figure S2. *DSC values on the test subset across different histological features at optimal epoch.*

3. Comparison of model predictions with ground truth annotations

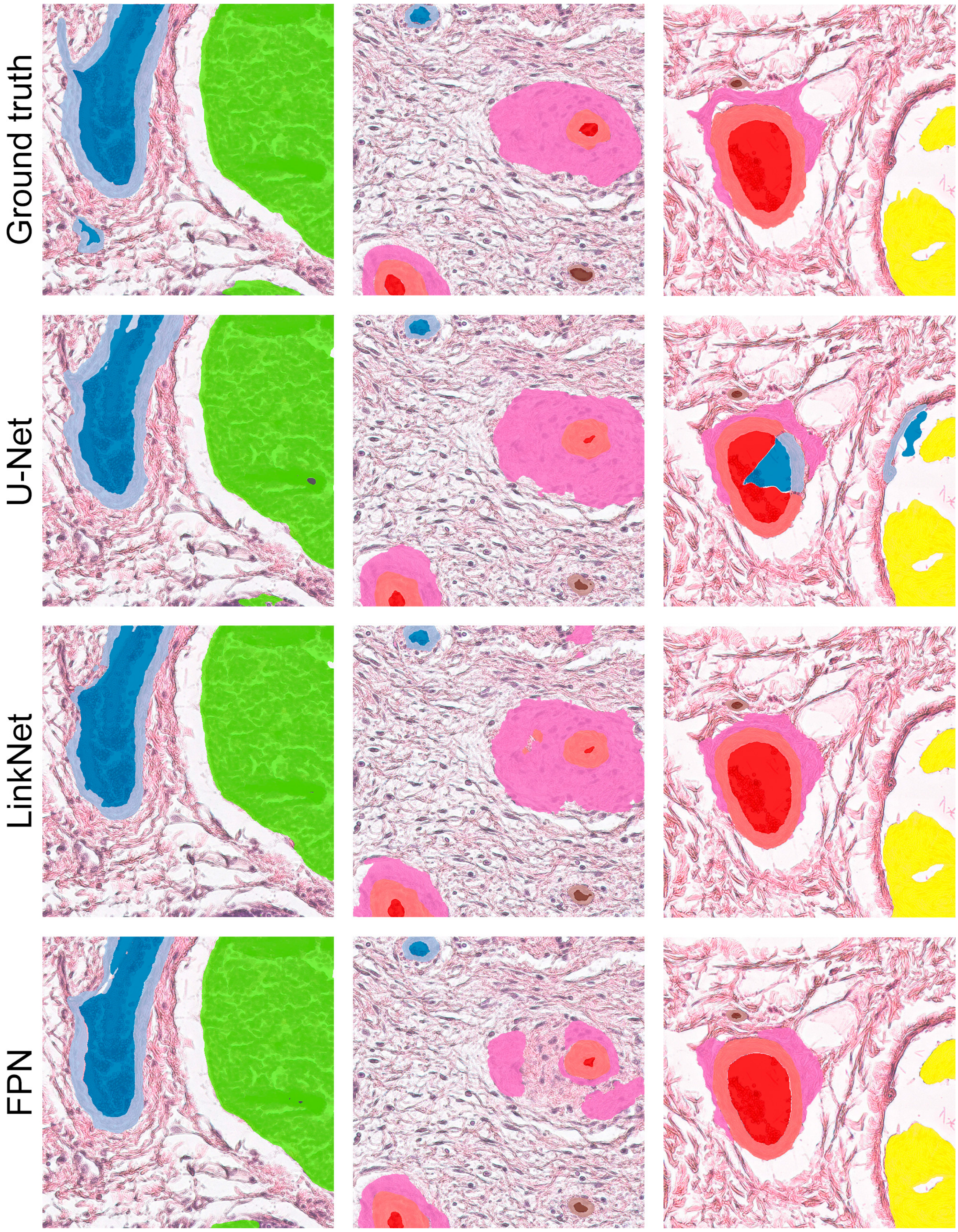

Figure S3. *Comparison of U-Net, LinkNet, and FPN predictions with ground truth annotations.*

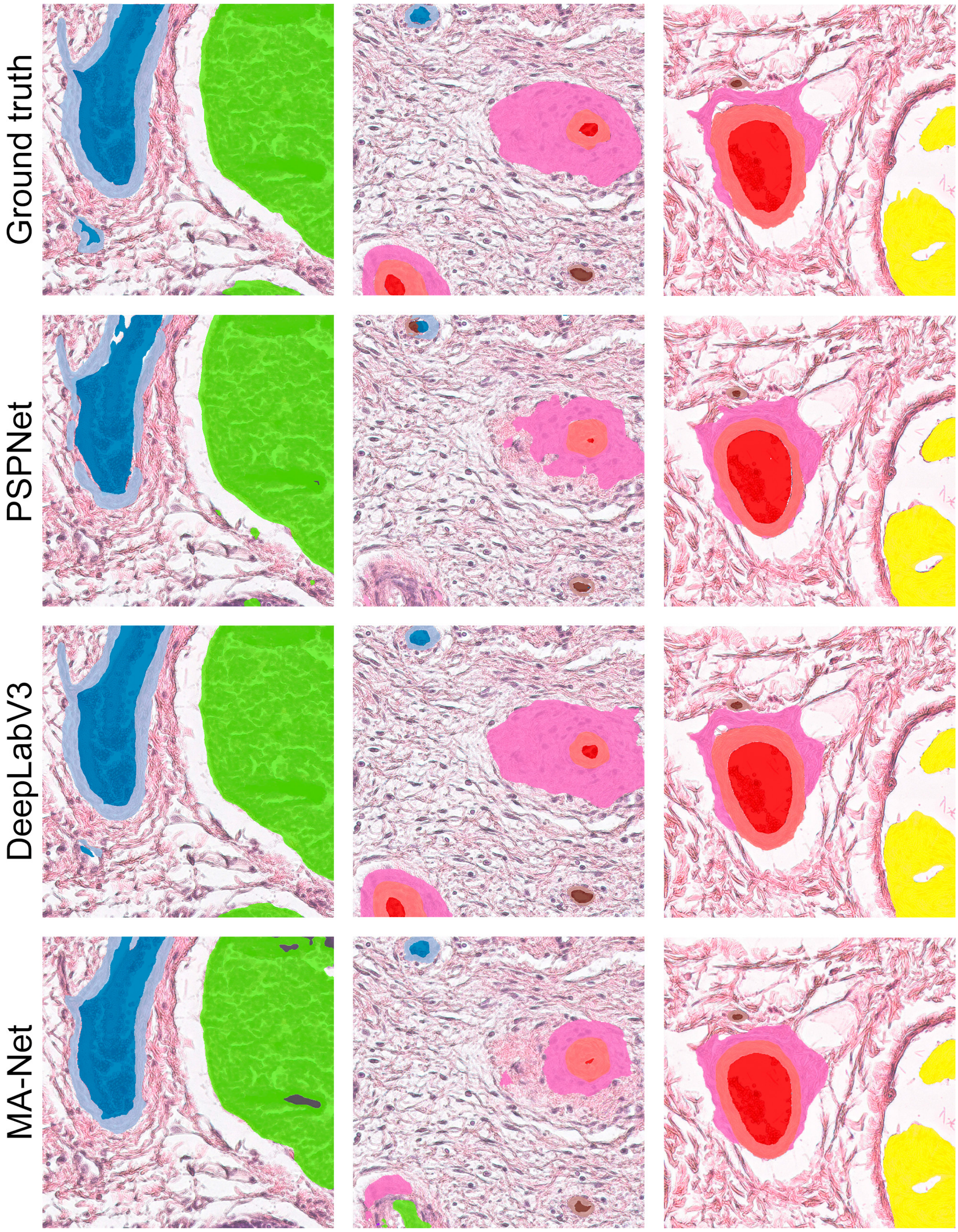

Figure S4. *Comparison of PSPNet, DeepLabV3, and MA-Net predictions with ground truth annotations.*
